# Supplementary material for: Vitamin D Modified DSS-Induced Colitis in Mice via STING Signaling Pathway
Source: Biology (Basel). 2025 Jun 18;14(6):715. doi: 10.3390/biology14060715 (PMC12190092; doi:10.3390/biology14060715)
Supplement: Supplementary file 1 [file biology-14-00715-s001.zip › Supplementary Materials S1: 16s-report of gut microbiota/Groups/diff_analysis/UnivarTestGMM/figure/boxplot/top10.pdf]

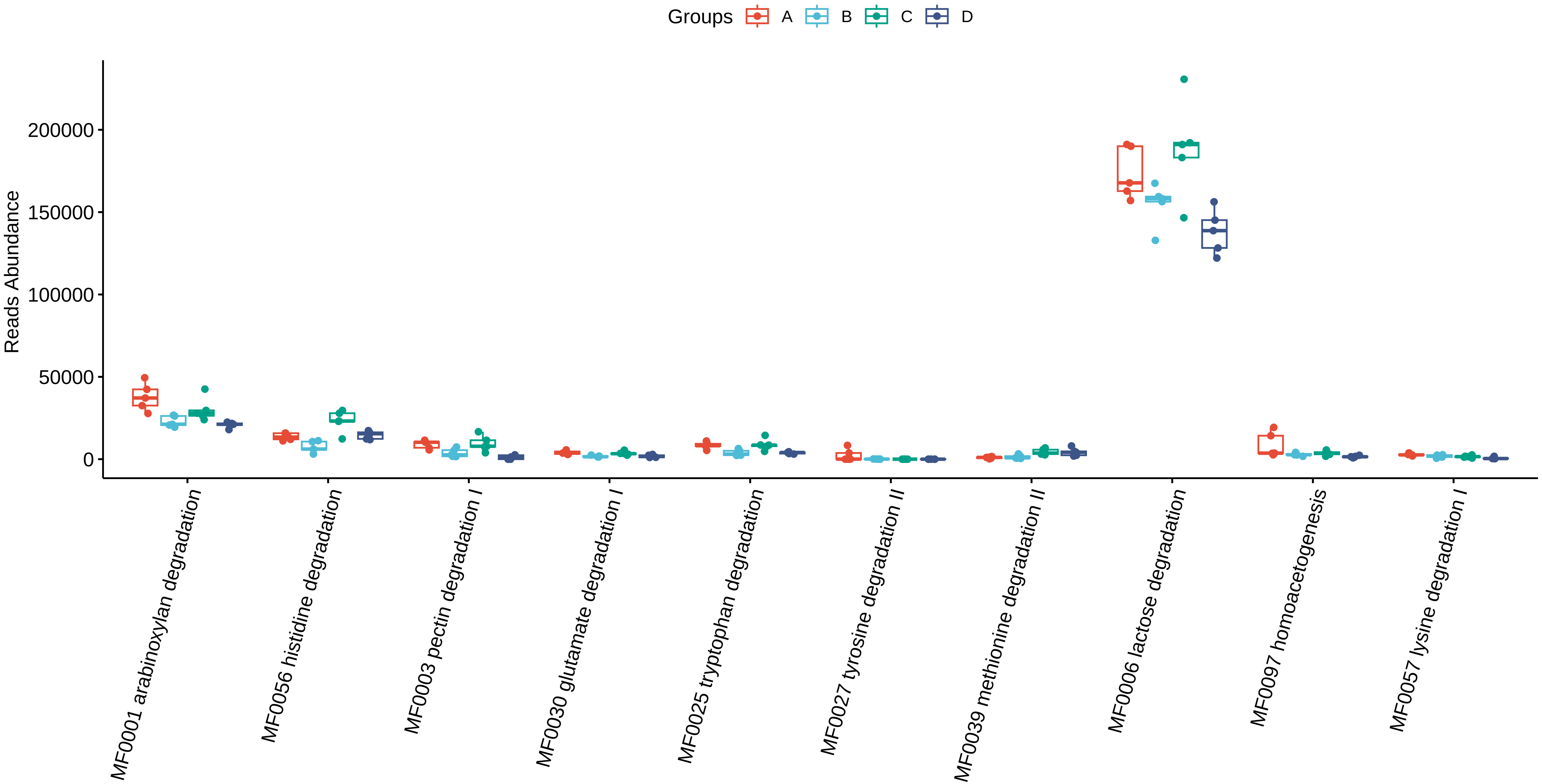
Groups
A
B
C
D
Reads Abundance
200000
150000
100000
50000
0
MF0001 arabinoxylan degradation
MF0056 histidine degradation
MF0003 pectin degradation I
MF0030 glutamate degradation I
MF0025 tryptophan degradation
MF0027 tyrosine degradation II
MF0039 methionine degradation II
MF0006 lactose degradation
MF0097 homoacetogenesis
MF0057 lysine degradation I
